# Are Gap and Cast Indices Predictors of Efficacy of Reduction in Fractures of Both Bones of the Leg? A Cohort Study

**DOI:** 10.5704/MOJ.1807.003

**Published:** 2018-07

**Authors:** K Shalabh, S Ajai, K Vineet, A Sabir

**Affiliations:** Department of Orthopaedics, King George’s Medical University, Lucknow, India

**Keywords:** gap index, cast index, non-operative management, tibia and, fibula

## Abstract

**Introduction:** Non-operative management has successfully been practised for long in diaphyseal fractures of both bones of the leg. This study attempts to establish an acceptability criteria for plaster cast in order to predict future loss of reduction and its adequacy.

**Materials and Methods:** A total of forty subjects were included as per inclusion-exclusion criteria. Gap and cast indices were calculated in the immediate post reduction phase and at third week follow-up visit.

**Results:** The mean values of gap and cast indices in the immediate post-reduction phase were 0.35±0.220 and 0.99±0.08 respectively and at the third week follow-up the mean value for both the parameters in those without loss of reduction were 1.11±0.50 and 1.03±0.09 respectively and in those with loss of reduction were 0.84±0.44 and 1.01±0.06 respectively.

**Conclusion:** Gap and cast indices are not informative in assessing adequacy of reduction in diaphyseal fractures of both bones of the leg.

## Introduction

Closed fractures have been treated by reduction and casting since ancient times. The technique has evolved over a period of time and has now become more precise with better clinical and functional outcomes and fewer major side effects. Casting has been effectively used for almost all limb fractures but in particular has wide acceptance in forearm and leg fractures. Although major advances have been made in this regard, an accepted parameter to assess the functional and radiological outcomes of the casting techniques in lower limb fractures is still awaited. This eventually leads to certain patients being operated upon who could have been treated effectively by non-operative means. As such, there is a need to standardise the assessment of casting techniques to determine which patients would actually need operative intervention from those who were being managed without an operation, in order to avoid unnecessary complications and without compromising functional outcomes.

In the recent past many studies have been conducted to develop an index that could predict at the outset, the chances of failure of a particular cast. Amongst them, gap index and cast index are two such indices that have been proven to show a significant level of sensitivity and specificity in predicting outcomes of casting techniques in paediatric forearm fractures and reports have shown the efficacy of these indices in such fractures^1-3^. The causes of failure of a cast to hold reduced fracture segments can be many, ranging from a non-anatomical reduction^1,4, position of the limb after manipulation5^,^6^, inclusion or exclusion of adjacent joints in the plaster^7^, pre-manipulation displacement^1,8-9^ condition of soft tissue status and the seniority and experience of the surgeon performing the procedure^10^.

This study was conceived to evaluate the gap and cast indices as predictors of efficacy of plaster cast in the management of displaced diaphyseal fractures of both bones of the leg, as in this part of country due to poverty and excessive load on operation theatre, many simple fracture both bones of the leg are being routinely managed by closed reduction and above knee plaster cast.

## Materials And Methods

With approval of the departmental ethical committee (137/OS/06/2014) the present prospective cohort study was carried out between July 2014 and December 2017. This open ended study was conducted on the patients presenting to the Orthopaedic Out Patients Department and Trauma Centre of our institution. All adult patients of either gender presenting within seven days of injury with closed displaced diaphyseal fractures of the both bones of the leg and considered suitable for non-operative treatment were included in the study. All those with more than one fracture in the same limb, unsuitable fractures pattern (comminuted, compound and segmental fracture) not suitable for conservative management, pathological fractures, open fractures, and single bone fracture were excluded from the study.

**Fig. 1:**
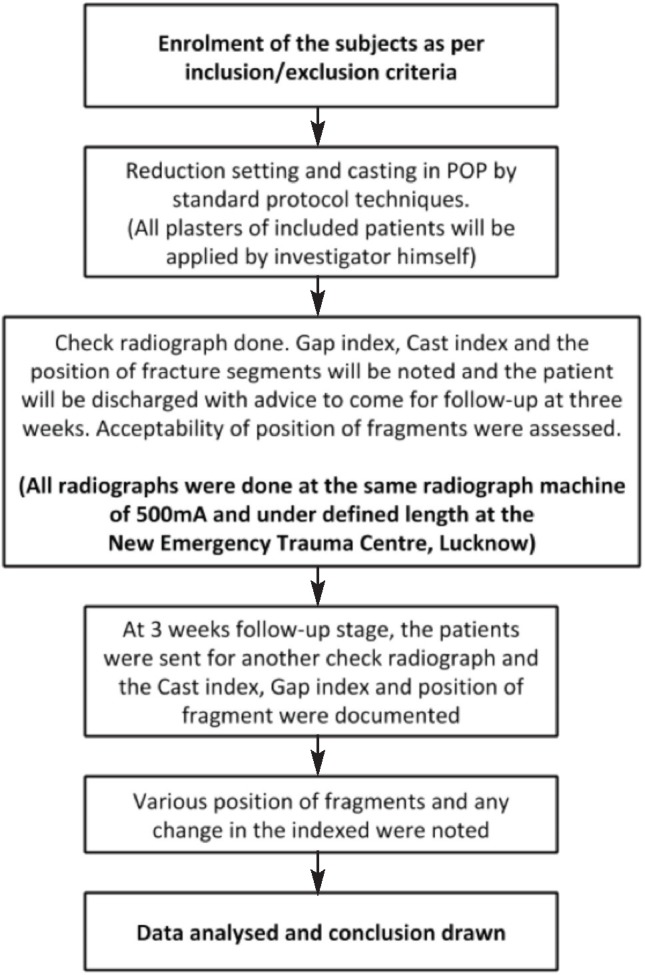
Flowchart of methodology.

**Fig. 2:**
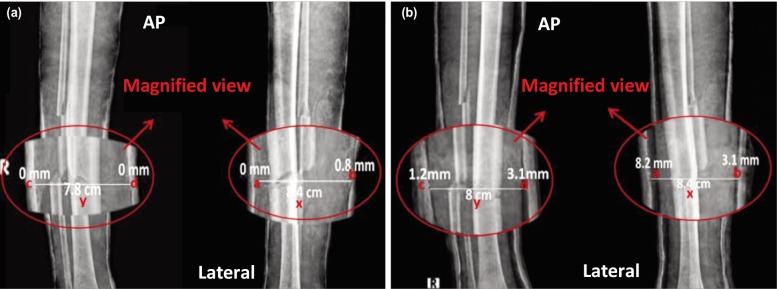
Radiographs of fracture site (a) Immediate post-reduction and (b) At 3 weeks follow-up.

After obtaining written informed consent from the patients with the procedures explained, the cases were recruited into the study and basic demographic parameters recorded. Fractures were reduced under general anaesthesia under image intensifier. Check radiographs (AP and lateral views) were taken to measure post reduction gap and cast index. All patients were discharged after 48 hours and called for follow-up after three weeks and the same parameters were assessed. As per evidence available for forearm fractures (distal end radius)^6^, we considered cast index 0.8 and sum of gap index 0.15 as threshold indices and utilised these values for our interpretation and analysis (Fig. 1). For fracture reduction, no angulations of more than 5 degrees of varus/valgus and 10 degrees in antero-posterior plane were accepted. The accepted criteria were fractures with at least 50% cortical contact in both views, with neither over-riding of fractured fragments nor rotation (Fig. 2).

**Fig. 3:**
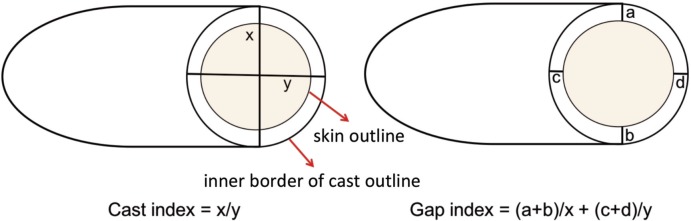
Measurement of cast index and gap index.

The cast index was calculated by measuring the inside diameter of the plaster in the lateral view as a ratio of the diameter in the antero-posterior view at the level of fracture/moulding site. The gap index was a measure of the space between the plaster and the skin measured as a ratio of the inside diameter of the plaster at the level of the fracture/moulding site (Fig. 3). On the radiographs, this was the radiolucent space between the plaster cast and the less dense but easily identifiable outline of the skin. It was measured at the fracture level in both the antero-posterior and the lateral views. The sum of the two measurements was calculated to obtain the gap index. Sensitivity, specificity, positive and negative predictive values, accuracy and odds ratio of the cast index and the sum of gap index were determined as predictors of failure of cast. The measurement was taken at the level of fracture of tibia in both bones were fractured^2,6,11-12^.

x= Distance between inner border of the plaster/cast at the fracture/moulding site in lateral view.

y= Distance between inner border of the plaster/cast at the fracture/moulding site in AP view.

a= Distance between identifiable outline of skin and inner border of the plaster/cast at the fracture/moulding site (Anteriorly in AP view)

b=Distance between identifiable outline of skin and inner border of the plaster/cast at the fracture/moulding site (Posteriorly in AP view).

c= Distance between identifiable outline of skin and inner border of the plaster/cast at the fracture/moulding site (Medially in lateral view).

d=Distance between identifiable outline of skin and inner border of the plaster/cast at the fracture/moulding site (Laterally in lateral view).

Statistical analysis was performed using GraphPad Prism for Windows program version 7.0. Categorical variables was presented in numbers and percentage (%) and continuous variables were presented by mean±SD value when required. For comparison of the means between the two groups, analysis by Student’s t-test (unpaired) with 95% confidence interval was used. A p<0.05 or 0.001 was regarded as significant.

## Results

A total of 40 patients were recruited for the study. The demographic details are given in Table I. At three weeks follow-up, reduction was lost in seven cases and maintained in 33 cases. The mean value of gap and cast indices in the study population were calculated in the immediate post-reduction period and at three weeks follow-up (Table II). The mean values of all subjects in the immediate post-reduction phase and at three weeks follow-up period in those with loss and without loss of reduction were analysed (Table II). On statistical analysis of the cast and gap indices, we found no significant difference between the subjects at three weeks follow-up in whom reduction was lost and in those where reduction was not lost (p-value: 0.1943 and 0.5794 respectively).

**Table I:** Subject demographics and location of fractures

Parameter	Frequency
Age	
18-65 years	40
Leg	
Left	18/40 (45%)
Right	22/40 (55%)
Sex	
Male	26/40 (65%)
Female	14/40 (35%)

**Table II:** Mean comparison of Gap index and Cast index with reduction loss and without reduction loss in both bones of the leg

	Both bone leg (N=40) Post reduction (mean±SD) N= 40	Follow-up without reduction loss (mean±SD) (N=33)	Follow-up with reduction loss (mean±SD) (N=7)
Gap index	0.35±0.22	1.11±0.50	0.84±0.44
Cast index	0.99±0.08	1.03±0.09	1.01±0.06

## Discussion

An operative intervention brings with it a myriad of factors and complications into play along with the imposition of inevitable financial, psychological and physical strain on the patient. Various studies had been done in this respect to predict values for gap index and cast index in patients treated by closed manipulative reduction and cast application which could be used universally immediate post-reduction to predict outcome of fractures, but these studies were limited only to distal forearm fractures of paediatric age group^6^.

Bhatia *et al* studied distal forearm and wrist fractures in a paediatric age group and concluded their cut-off value of cast index of >0.80 implying that those having a cast index of >0.80 had greater chances of fracture displacement in subsequent follow-up^3^. We, at our institute, have been treating diaphyseal fractures of both bones of the leg for a long time, with closed reduction and plaster cast with good results. We do perform surgical intervention in cases for selected indications as we have a high turnover of trauma cases.

In our study, we included diaphyseal fracture of both bones of the leg in an adult population for the evaluation of gap index and cast index as predictors of efficacy of cast in non-operative management. On comparing the relationship between gap and cast indices at three weeks of those cases in whom reduction had failed and those in whom it was contained, we found no statistical difference in results and we were not able to have any cut-off value owing to the fact that the patients followed up were not showing any significant trend in either group. This was probably because of the fact that the basic structural anatomy is different in distal forearm and mid-leg region. The wrist or the distal forearm is relatively flat antero-posteriorly with an elliptical cross section at that level, whereas the leg region in diaphyseal area has a circular cross section. The gap and the cast indices therefore could not provide the same information as what they provide in distal forearm fractures. Moreover the values of gap and cast indices which we obtained from both the group of patients at three weeks follow-up were also very aberrant.

Although there was no statistical significance it was observed that in our study population subjects with a gap index of >0.34 have lesser chances of loss of reduction and similarly subjects with a cast index of >0.99 have higher chances of loss of reduction. These observations definitely need better evidence backed by a large population size, which was our major limitation.

## Conclusion

Parameters (gap index and cast index) in contrast to their appropriateness in assessment of reduction in distal radius fractures (metaphyseal) are not the appropriate tool in assessment of adequacy of reduction in diaphyseal fracture of both bones of the leg in an adult population.
